# Diclofenac toxicity in susceptible bird species results from a combination of reduced glomerular filtration and plasma flow with subsequent renal tubular necrosis

**DOI:** 10.7717/peerj.12002

**Published:** 2021-08-23

**Authors:** Bono Nethathe, John Chipangura, Ibrahim Zubairu Hassan, Neil Duncan, Emmanuel Oluwasegun Adawaren, Lauren Havenga, Vinny Naidoo

**Affiliations:** 1 Department of Paraclinical Science, University of Pretoria, Onderstepoort, Pretoria, South Africa; 2 Department of Food Science and Technology, University of Venda for Science and Technology, Thohoyandou, Limpopo, South Africa; 3 Department of Pathology, University of Pretoria, Onderstepoort, Pretoria, South Africa; 4 Department of Anatomy and Physiology, University of Pretoria, Onderstepoort, Pretoria, South Africa

**Keywords:** Diclofenac, Toxicity, Organic anion transporters, Multidrug resistance protein, Chicken, Vulture

## Abstract

Diclofenac caused the death of millions of vultures on the Asian subcontinent. Other non-steroidal anti-inflammatory drugs (NSAIDs) have since also been shown to be toxic to vultures with the exception of meloxicam. For this study, we evaluated the effect of diclofenac on renal uric acid transport and glomerulus filtration in an acute toxicity model. In a two-phase study with the same birds, healthy chickens (a validated model species) were treated intravenously with para-amino hippuric acid (PAH) and iohexol (IOH) in combination in phase 1. In phase 2, the same PAH and IOH combination was then combined with diclofenac (10 mg/kg). In both phases, blood and faeces were sequentially collected. In phase 1, the birds showed no signs of ill health. Moreover, PAH, IOH and uric acid clearance was rapid. In phase 2, two chickens showed early signs of hyperuricemia 8 hours after exposure and died approximately 24h later. Necropsy showed classic signs of renal damage and gout. Diclofenac had a rapid plasma half-life of elimination of less than 2 hours indicating that toxicity was likely due to an irreversible destruction of a physiological process. All the birds in phase 2 had decreased uric acid, PAH and IOH clearance in comparison to phase 1. The decrease in PAH clearance was variable between the birds (average of 71%) but was near 98% reduced in the two birds that died. It is concluded that diclofenac alters both renal perfusion and renal plasma flow, with death associated with tubular secretion being reduced to negligible functionality for a prolonged period. This would support previous *in vitro* findings of early cell death from ROS accumulation. However, further evaluation is needed to elucidate this final step.

## Introduction

Old world vultures are purely scavengers reliant on carrion for their nutritional needs. For this adaptation, they are large sized for optimal soaring and energy expenditure; have excellent eyesight; and a high capacity haemoglobin function (Wink, 1995; Rodrigues, 2007). A notable adaptation is their large oesophageal diverticulum known as a crop. The crop represents a temporary storage area with capacity circa 20% of body weight. The crop allows for single large meal consumption and thereafter slow release to the proventriculus, longer than other raptors (Kierończyk et al., 2016). This feeding behavior, unfortunately, does predispose the bird to toxins in the food, as evident in Asia. Vultures on the Asian subcontinent have in the recent past been brought to near extinction through their inadvertent exposure to diclofenac in their food (Oaks et al., 2004; Swan et al., 2006a), with a resultant overall population decline of 99%. Species most effected were the white-rumped, long-billed and slender-billed vulture (*Gyps bengalensis*, *G. indicus* and *G. tenuirostris*) (IUCN, 2017).

Modelling of exposure suggests that toxicity resulted from an intake of 1 kg of meat contaminated with diclofenac equating to a dose of 0.8 mg/kg (Green et al., 2004). Clinical signs seen were a depressed habitus, inappetence and drooping of the head at 24 h post exposure, and death after 48 h. Pathological changes were marked hyperuricaemia and severe visceral gout (Meteyer et al., 2005). Since its description in the Asian vulture species, sensitivity has been confirmed in the Cape vulture (*G coprotheres*), African white backed vulture (AWBV) (*G. africanus*), Eurasian griffon vulture (*G. fulvus*) and the Stepp eagle (*Aquila nipalensis*) (Swan et al., 2006a; Rattner et al., 2008; Naidoo et al., 2009; Sharma et al., 2014), but surprisingly not the Turkey vulture (*Cathartes aura*). In an unexpected manner, the chicken was also susceptible to the effects of the drug, albeit at a higher LD_50_ (10 mg/kg) (Naidoo et al., 2007). Since diclofenac was shown to be unsafe, a number of other NSAIDs, except meloxicam, have also tested as unsafe (Swan et al., 2006b).

While the pathological changes associated with diclofenac are well documented, the mechanism and apparent sensitivity of old-world vulture species remains unknown. Being predominantly carrion eaters, their natural exposure to higher uric acid from both the catabolism of purines and being uricotelic is expected to be contributory (Long & Skadhauge, 1983; Berger, Yü & Gutman, 1960; Dudas et al., 2005). It has, however, been speculated that diclofenac interferes with active excretion of uric acid at the proximal convoluted tubules (PCT) (Naidoo & Swan, 2009), or by interfering with blood supply to the cranial lobe (Meteyer et al., 2005). An *ex vivo* study with limited vulture tissue showed inhibition of para-aminohippuric acid (PAH) transport, indicative of organic anionic (OAT) channel inhibition (Naidoo & Swan, 2009). This is not unexpected as NSAIDs, including diclofenac, are OAT inhibitory in people (Sekine, Cha & Endou, 2000).

The aim of this study was to further expand the knowledge on the pathophysiology of diclofenac toxicity in vultures. To achieve this, the influence of diclofenac on uric acid excretion, glomerular filtration and plasma flow was evaluated in a domestic chicken model, previously validated as a suitable model for diclofenac toxicity studies (Naidoo et al., 2007). In this previous study, it was demonstrated that the time leading to toxic effects, clinical signs and pathological changes seen with diclofenac are identical between the chicken and the numerous vulture species (Naidoo et al., 2007). The other reason for using a surrogate species is the highly endangered status of vulture species (IUCN, 2017). More specifically, for this study chickens were exposed to diclofenac in the presence of PAH and iohexol (IOH) (Omnipaque) as per the model of Laroute et al. (2005). The former is a very specific transport substrate of the OAT channel while omnipaque is a non-radioactive radiographic marker of renal blood flow and glomerular filtration. Further, Smeets et al. (2004) showed that the Multiple Drug Resistance Protein (MRP) were not involved in PAH excretion in rat kidney slice cultures (Smeets et al., 2004). In combination, these two substances have been previously used in the dog as a means to non-lethally evaluate changes in renal functionality at the level of the glomerulus and the renal tubular basal transporters (Laroute et al., 2005).

## Materials and methods

### Animals welfare, treatment and sample collection

Before commencement of the study, ethical clearance (V108-16) was obtained from the animal ethics committee of the University of Pretoria. Six (6) female Ross Broiler chickens (*Gallus gallus*) were used in a two-phase study for the evaluation of renal plasma flow, glomerular filtration rate and uric acid clearance in the absence or presence of diclofenac (10mg/kg). The sample size was based on the previous toxicity study of Naidoo et al. (2007) in which the domestic chicken was deemed to be an appropriate model species.

The chickens were grouped-housed at the University of Pretoria Biomedical Research Centre (UPBRC) and fed commercial broiler foods and an antibiotic-free ration ad libitum. The chickens were kept on the floor with wood shavings as scratch and bedding material in an enclosure of 1.2 m × 2 m in size. On arrival as day-old chicks they were housed at a temperature of ±31 °C. The temperature was subsequently lowered by 1 degree every second day until 23 °C was reached. Humidity was allowed to fluctuate with ambient conditions. The room was under mechanical ventilation by a horizontal fan that ran for 30 s every hour as the minimum setting. This was increased steadily to 5 min every 60 min, as dictated by ambient temperature. The chickens were four-weeks old for the start of phase 1. The light-dark phasing for the study was 12/12 h. For the actual day of the study per phase and in order to facilitate easier handling and to minimize stress, the birds were kept in separate cages for the single day.

In phase 1 (control phase), birds were treated with a single intravenous dose of PAH (10 mg/kg) (Merck, South Africa) and iohexol (Omnipaque, 1.25 mg/kg) (GE Healthcare (Pty) Ltd, USA), mixed in one syringe immediately prior to administration. Faecal samples were collected at 0, 2, 6, 8 and 12 h and stored at −80 °C until further analysis. Blood was collected from the brachial or jugular vein when necessary at 2, 4, 6, 8, 12 and 24 h into a syringe and immediately transferred into a 5 ml lithium heparinised vacutainer (Becton, Dickson and Company, South Africa). Blood samples collected were centrifuged (Thermo Fisher Scientific, Germany) at 3,000× g at 4 °C for 15 min and the supernatant of each sample was transferred into labelled polycarbonate tubes and stored at −80 °C until further analysis. The birds were allowed a two-week recovery period before phase 2 started. In phase 2 the same birds as in phase 1, received a single dose of diclofenac (10 mg/kg) (Merck, South Africa). Two (2) hours after administration of diclofenac, the mixture of PAH and omnipaque was administered and the same methodology was applied as per phase 1 for sample collection.

Stored faeces and plasma samples from a previous study of two Cape griffon vultures (*Gyps coprotheres*) treated with meloxicam (2 mg/kg) were also analysed for uric acid concentrations as a control for species specific uric acid clearance (Adawaren et al., 2019). The faecal and plasma samples were selected for the 10 h time points, since plasma analysis showed both the absence of meloxicam (after 10 half-lives) and that uric acid concentrations were at pre-meloxicam exposure concentrations.

### Sample analysis

#### Diclofenac analysis in domestic chicken plasma samples

Diclofenac in plasma samples, were analysed by using a validated HPLC method as previously described (Naidoo et al., 2007). In brief, plasma samples (200 µl) were mixed with diethyl ether (400 µl), 0.3 M potassium dihydrogen phosphate (400 µl) and vortexed. The organic layer was separated in an ice bath (methanol/solid carbon dioxide), evaporated to dryness and dissolved in 400 µl mobile phase. Samples were analysed on a Beckman System Gold HPLC (Beckman Instruments, Fullerton, CA, USA). Separation was achieved with a C18 column (250 mm × 4.6 mm × 5 µm; Thermo Scientific, Runcorn, UK). The mobile phase consisted of 0.05 M sodium dihydrogen phosphate (pH  = 4.85 to 4.89):CH_3_CN, 42.5:57.5. One hundred (100) µl of the reconstituted samples was injected onto the column at 1 ml/min in an isocratic run. Detection of diclofenac was carried out at 275 nm. The total runtime per sample was 8 min with retention times of 4.9 min. Control values (100–0.39 µg/ml) showed a mean accuracy of 99%. When sufficient time points were available, samples were analysed by non-compartmental pharmacokinetic analysis (Thermo Fisher Kinetica 5.1, USA) using the standard settings.

#### Diclofenac analysis in domestic chicken faecal samples

For validation, 25 mg of faeces (the white urine and solid components mixed in natural proportions) were spiked with 200 µl of diclofenac standard concentrations (6.25 to 200 µg/ml) in triplicate. One millilitre of acetonitrile was added to the spiked excreta sample and homogenised. The homogenate was centrifuged at 10,000 rpm for 10 min at 4 °C. After centrifugation, the supernatant was filtered into a clean marked glass tube and evaporated to dryness at 60 °C under nitrogen. This was followed by adding 200 µl of the mobile phase (acetonitrile: sodium dihydrogen phosphate; 50:50) into the dried sample and vortexing for 2 min to mix. 30 µl of the reconstituted sample was injected onto the HPLC column, using the same method and equipment as above, with the exception of the mobile phase. After optimizing, acetonitrile: sodium dihydrogen phosphate; 40:60 yielded clearer separation of peak.

The elimination constant and half-life of elimination was determined from cumulative excretion over time period of evaluation. For this calculation, the total concentration of diclofenac for each time period of measurement was converted to the ARE (amount remaining to be excreted). The ARE was the total cumulative amount excreted over the total period, less the total amount excreted per time point in an iterative manner starting from the first time point of measure. The slope (Kel) of the ARE *versus* time period of collection on the logarithmic scale was the elimination constant. The half-life of elimination was determined as Ln(2)/Kel. Renal clearance was measured by dividing total faecal excretion of uric acid, divided by total time period of evaluation, divided by drug concentration in plasma (Gibaldi & Perrier, 1982).

#### PAH and iohexol analysis in domestic chicken plasma samples

The samples were analysed at the commercial PharmOVS Parexel laboratory in Bloemfontein, South Africa using a method developed and validated by the laboratory. Samples were thawed in a water bath (Agilent Technologies, Inc, USA) at ∼22 °C and vortexed briefly. The samples were centrifuged for 5 min at 1,300× g and 50 µl of plasma was aliquoted into microcentrifuge tubes. 950 and 400 µl of methanol was added to the samples containing internal standards 250 ng/ml (Diphenhydramine (N-[2-(benzhydryloxy)ethyl]-N,N-dimethylamine)) and 5 ng/ml (Acetaminophen (N-(4-hydroxyphenyl)acetamide)) for IOH and PAH respectively. The samples were vortexed for 30 s and centrifuged for 5 min at 500× g. The supernatant (100 µl) was transferred into a microcentrifuge tube and diluted with 0.1% formic acid (1:8; 1:3; v/v) for PAH and IOH, respectively. The extracts were transferred to a 96-well plate.

Samples were analysed by Liquid chromatography tandem mass spectrometry (LC/MS/MS) using an Agilent 1,100 series high pressure liquid chromatograph (Agilent Technologies, Inc, USA) with temperature controlled autosampler (model G1367B) and a diode array detector, coupled to a Sciex API4000 QTRAP mass spectrometer and fitted with a “Turbo V” electrospray ionization (ESI) source. The analytical column used was Supelco Discovery column (C18) column (150 × 2.1 mm, 4 µl particle size) fitted with a phenomenex security guard system containing C18 (4 × two mm) pre-column. The mobile phase consisted of methanol: 0.1% formic acid solution (1:1, v/v). The pump delivered the mobile phase at the flow rate of 250 µl/min. The sample injection volume used was 4 and 3 µl for IOH and PAH, respectively. The autosampler was equipped with a 96-well plate tray and it was fitted with a cooling device to keep the samples at ∼5 °C. The retention times were as follows: PAH at ∼1.70 min, IOH at ∼1.67 min and both internal standards at ∼1.80 min.

The clearance of IOH (CL_IO_) and PAH (CL_PAH_) was determined using standard pharamacokinetic equation of Dose/Area under curve (AUC_last_), where AUC_last_ was determined using the linear trapezoidal rule for the visible portion of the plasma concentration *versus* time curve. The tubular clearance of PAH (Tm_PAH_) was determined as CL_PAH_ –CL_IO_, CL_IO_ representing glomerular filtration and CL_PAH_ the total PAH clearance.

#### Uric Acid analysis in domestic chicken and Cape vulture plasma samples

Plasma uric acid concentrations were determined by a commercial veterinary clinical pathology laboratory using a Cobas Integra 400 plus analyzer (Roche, USA) with an autoinjector. The method was routinely quality tested and validated by the laboratory (University of Pretoria, South Africa) using available standards. The method more specifically, reacts uric acid in the sample with uricase to produce allantoin and H2O2. The formed peroxide reacts with peroxidase (POD) and 4-aminophenazone (TOOS) to form quinoneimine dye. The intensity of the red colour formed is then proportional to the uric acid concentration in the sample, and quantified at an absorbance at 552 nm, with the final automated results provided by the machine. The total exposure to uric acid to the last time point was determined as area under curve (AUC_last_-UA) using the liner trapezoidal rule.

#### Uric acid determination in domestic chicken and cape vulture faecal samples

The uric acid concentration in faeces was determined using the method of Marquardt (1983). The total faeces collected per period was dried in an oven. Ten mg of the faeces was grounded and added to 10 ml of 0.1 M glycine buffer, pH 9.3 and extracted at 40 °C with constant mixing for 1 h. The suspension was allowed to settle, and an aliquot was diluted 15-fold with 14 volumes of 5.35% perchloric acid (PCA). The samples were centrifuged at 20.000× g for 5 min. The absorbance of the samples was determined at 285 nm at a path-length of one cm. The concentration of uric acid in the faecal sample was determined against a standard curve for uric acid (MW, 168.1). For the standard curve a known concentration of uric acid was dissolved into a 0.1 M glycine buffer, PH 9.3. The standard curve was diluted with different amounts of water, prior use, with equal volume of 10% PCA. Absorbance was determined against the appropriate blank at the same wavelength as the unknown samples (285 nm). Cloacal faecal samples from untreated chickens obtained opportunistically post-mortem were used to establish the standard curve. The total uric acid concentrations in the faeces were used to determine uric acid clearance with Cl_UA=_ Total Faecal Uric Acid/Plasma Uric acid for the same time point. The faecal clearance hereby calculated becomes a reasonable estimate of urinary clearance as previous studies have shown that nearly 98 to 99% of uric acid in the faeces is of urinary origin and the remainder of cloacal origin (Beck & Chang, 1980), *i.e.,* the cloacal samples used in establishing the standard curve allows for this 1% correction. T_urate_ was calculated as the clearance of IOH, multiplied by the plasma uric acid concentration at 12 h, subtracted from total excreted uric acid over the preceding 4 h. The difference in the applicable parameters was compared between phase 1 and 2 by means of a paired *t*-test after confirmation of data normality on natural logarithmic transformed data (IBM SPSS Statistics 27, South Africa).

### Radiographic analysis

Five minutes following the administration of IOH, all chickens were radiographed and visually evaluated to ascertain if the different lobes of the kidneys were visible. Radiographs were taken using a diagnostic radiographic unit and a computed radiographic system on a Carestream digital analyser (UPBRC).

### Pathology and histopathology

All chickens, either 48 h after treatment or unscheduled deaths were subject to full necropsy. Chickens were euthanized with a pentobarbitone overdose (200 mg/kg). Necropsy was conducted and lesions seen were recorded. For histopathology, tissue samples collected for residue analysis (kidney, liver, heart, spleen, intestine and lungs) were preserved in 10% buffered formalin. Samples preserved in formalin were trimmed, embedded in paraffin, sectioned and stained using standard methods with hematoxylin and eosin (Naidoo et al., 2007).

### Vulture feeding study

The use of vultures, a CITES protected species was approved by the South African Department of Environmental affairs (07140). The White-back vultures (*n* = 11) were housed in-doors at the UPBRC in individual cages and had free access to water. Blood samples were collected from vultures at 4, 12 and 24 h *via* the wing vein after feeding of 200 g of beef. The collection points were kept to a minimum to prevent regurgitation which is a natural defence mechanism in vultures. The uric acid plasma concentrations in the vultures were evaluated as above. Clearance was determined using standard methods, with area under the uric acid curve determined using the linear trapezoidal rule following baseline correction for endogenous uric acid production. The dose of uric acid was determined from the table of Kaneko et al. (2014) which indicates 162 mg of uric acid per 100 g of topside.

## Results

### Histopathology

No ill-health was observed in chickens in phase 1. However, in phase 2, two chickens (9192 and 9194) died while the remaining four chickens survived until the termination point. The chickens that died were diagnosed with severe renal-glomerulo-tubular necrosis and urate nephropathy. The chickens had dilation of mammalian glomeruli and some renal tubules, with complete loss of internal structure and only the basement membrane remaining. Multifocal renal tubules in between were filled with urate deposits, with complete loss of tubular epithelium. The urate deposits were surrounded by multinucleated giant cells and macrophages. For the chickens that survived all the organs were normal except the lungs and one chicken (9189) had early signs of urate nephropathy, albeit less severe than the chickens that died (Figs. S1– S4).

### Pharmacokinetics

After administration of a single dose of diclofenac (10 mg/kg, LD_50_) to 6 domestic chickens by intravenous route, four of the treated chickens, had barely detectable diclofenac concentrations at the first sampling point (0.29 ± 0.1395 µg/ml; first sampling was 4 h after diclofenac administration) which was below the lower standard tested (0.4 µg/ml) and completely non-detectable thereafter. Two of the latter chickens died within 48 h. Guided by the above findings, the half-life elimination was much shorter than 2 h. For the remaining two chickens (9189 and 9190), diclofenac concentrations were detectable at a level which allowed for pharmacokinetic analysis (Fig. S5). The half-life, AUC_last_ and V_d_ were 2.4 ± 1.8 h, 10.79 ± 2.55 µg/ml*h and 3.65 ± 3.27 l/kg respectively (Table 1).

**Table 1:** Pivotal pharmacokinetics parameters for diclofenac following intravenous administration in domestic chicken plasma using a one compartmental analysis.

Pk parameters	Units	Bird 9189	Bird 9190	Mean	SD
C_max_	µg/ml	1.46	3.95	2.705	1.76.696
T_max_	h	2	2	2	0
Vz (V_d_)	l/kg	5.9613798	1.34169117	3.651535485	3.266613
Lz (ke)	h^−1^	1 0.1868	0.592	0.3894	0.28652
T1/2	h	3.71063801	1.17085672	2.440747365	1.795897
AUC_last_	µg/ml*h	8.98	12.59	10.785	2.552655
AUC_ext_	µg/ml*h	1.23126338	0.625	0.92813169	0.428693
AUC_inf_	µg/ml*h	10.2112634	13.215	11.7131317	2.123963
%AUC	%	87.9421053	95.2705259	91.6063156	5.181976
AUMC	µg/ml*h2	42.3	28.14	35.22	10.01263
Cl	l/kg*kg	1.11358575	0.79428118	0.953933465	0.225782

**Notes.**

C_max_ maximum concentration T_max_ time to maximum concentrations Vz (Vd) volume of distribution Lz (ke) lambda (elimination constant) T1/2 half-life AUC_last_ area under curve last AUC_ext_ area under curve extension AUC_inf_ area under curve infinity %AUC area under curve percentage AUMC area under movement curve Cl clearance

### Method validation and faecal diclofenac clearance

To ascertain the elimination constant of the drug, the chicken’s faecal samples were analysed for diclofenac concentrations. For the developed method, peak identification of faecal samples spiked with diclofenac was performed by comparing the retention time (4.9) with pure standard of diclofenac and confirmed by characteristic spectra obtained from the photodiode array detector, which also permitted the conformation of the purity of the peak (Fig. S6). The method was found to be linear at the range of 6.25–200 µg/ml for three independent curves with the co-efficient of determination (r^2^) above 0.99 (Table S1). The developed method had limit of detection (LOD) and limit of quantification (LOQ) values of 6.25 and 12.5 µg/ml respectively. After method validation faecal samples were analysed and the elimination rate constant (kel) and half-life (h) of elimination for diclofenac was 2.31 ± 1.95 h and the Lz was 0.52 ± 0.34 h^−1^ (Table 2). Despite all the chickens having quantifiable half-life of elimination, there was poor correlation between the plasma and faecal half-life of elimination for chicken 9189 and chicken 9190.

**Table 2:** PK parameters of diclofenac concentration in the chicken faecal samples.

Animal	Weight (kg)	Clearance (l/kg*kg)	Kel (h^−1^)	Half-life (h)
9189	2.16	0.16	1.04	0.67
9190	2.99	0.14	0.60	1.16
9191	2.80	0.27	0.15	4.57
9192	2.54	0.19	0.48	1.45
9194	2.67	0.51	0.14	5.02
9195	2.92	0.30	0.70	1.00
Average	2.68	0.26	0.52	2.31
SD	0.30	0.14	0.34	1.95

**Notes.**

Kel (Lz), elimination constant.

### Plasma and faecal uric acid, PAH and iohexol clearance

**Figure 1:**
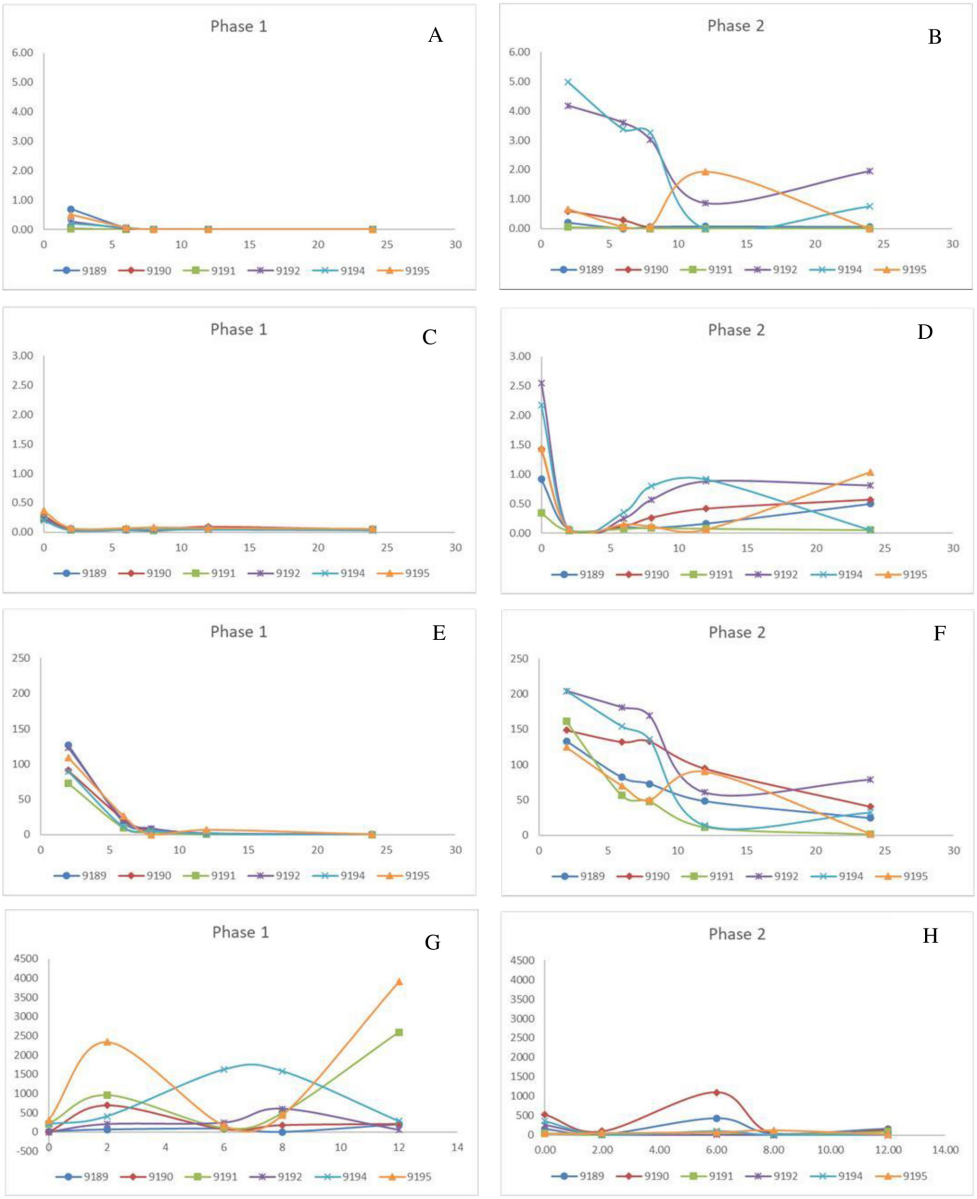
Change in pivotal parameters in chickens before (1) and after exposure to diclofenac (2) over time (h). (A and B) p-aminohippuric acid (µg/mL); (C and D) Plasma uric acid (mg/mL); (E and F) Iohexol µg/mL; (G and H) Total excreted uric acid (mg).

No changes in plasma uric acid concentrations were noted in phase 1 for any time point monitored, including before and after PAH administered, which indicated unconstrained excretory capacity. After the administration of diclofenac, plasma uric acid concentrations increased rapidly over a period of time, even though the concentrations at the start of each phase were within the normal ranges (0.217–0.403 mmol/l). The change in plasma uric acid for phase 2 was evident as early as 2 h post exposure, and for most chickens had peaked by 8 h (Times based on PAH/IOH administration, which was 2 h after diclofenac administration) (Fig. 1). The chickens (bird 9192 and 9194) that died 24 h after dosing had very high levels of plasma uric acid, which was more than 10 times the basal concentration as well as a more prolonged increase in uric acid concentrations (Fig. 1, Table 3). Concurrent to the plasma uric acid increase, faecal uric acid concentrations of the treated chickens and plasma uric acid clearance decreased, compared to their untreated values, with four diclofenac treated chickens showing up to a 99% decrease (Table 3), *i.e.,* the increase in plasma uric acid occurred concurrently with a decrease in the uric acid clearance. Of the four chickens in which the excretion of uric acid had decreased, two had died. For these two chickens the uric acid faecal concentrations were reduced to almost 0. While Tm_uricacid_ had decreased for all chickens, it was virtually absent in three chickens including the two that died (Table 3). As for diclofenac concentrations, there was poor correlation between plasma and faecal changes. The area under the curve last (AUC_last_) showed that the chickens that died had, at minimum, a two-fold higher total exposure to uric acid than the chickens that survived and a ten-fold increase to their healthy state. The cumulative result thus showed a strong relationship between death and absence of uric acid excretion, mainly at the level of the tubules.

For phase 1, PAH was not detectable in the plasma for two chickens (9190 and 9191) and for only two time points for the remaining four (9189, 9192, 9194, and 9195). As a result, it is concluded that PAH is rapidly cleared under normal conditions (Fig. 1). The clearance was estimated as presented in Table 3, using the LOQ as the first point value. The results were also highly variable in phase 2 between the chickens. The PAH clearance decreased from phase 1 to 2 with an average clearance of 32.34 and 8.99 L/h*kg respectively (Table 3). For five of the treated chickens, diclofenac induced an increase in the total exposure to PAH, as seen by the AUC_last_ (Table 3). The increase in PAH plasma concentrations was associated with a decrease in plasma clearance by 71.60 ± 16.54% (Table 3). For the two chickens that succumbed to the toxicity, PAH clearance decreased by 98% (Table 3). The plasma concentrations for IOH were more quantifiable (Fig. 1). The same trend was evident for IOH, however, with clearance decreased by up to 87%. The highest inhibition in the clearance of IOH was present in both chickens that died and for one that survived (Table 3). The tubular secretion of PAH (Tm_PAH_) was reduced by more than 90% for all the chickens except one bird which was reduced by 45%. Furthermore, it was also interesting to note that with the chickens that died the Tm_PAH_ was reduced by more than 98% (Table 3). In all cases the changes evident in phase 2 of the study were significant.

### Comparing uric acid levels of domestic chickens and cape vultures

Plasma uric acid concentrations of domestic chickens (untreated and treated) and cape vultures ranged from 0.202–0.543, 0.250–6.196 and 0.108–0.258 mmol/l respectively. As mentioned above, uric acid clearance of chickens treated with diclofenac significantly decreased over time, and at 12 h was 0.026 ± 0.41 ml/min while the control was 15.37 ± 19.01 ml/min. In comparison, the two vultures evaluated had uric acid clearance of 1.58 and 18 ml/min respectively. Considering that it is likely that there is diurnal variation and that the lower value may be indicative of a point of inactivity, at the upper end, the two species have similar clearance (Table S2). However, considering that the vultures were approximately 7.5 kg in body weight, their uric acid clearance by size was 2.41 ml/min/kg which is roughly half of that of the chickens at 5.71 ml/min/kg.

**Table 3:** Pivotal uric acid parameters calculated for the study and their associated change from the non-treated to the treated phase.

Animal	F_uric acid_ (mg)	P_uric acid_ (mmol/L)	Cl_uric acid_ (l/kg*kg)	AUC_uric acid_ (mg/L)	Cl_PAH_ (mg/min)	CL_iohexol_ (mg/min)	Tm_PAH_ (mg/min)	Tm_urate_ (mg/h)
9189	208.48/161.56(22.5)	0.3/2.98(1002)	1.97/0.18(0 )	568/2123(3.73 )	4.48/6.29(90.86 )	0.13/0.04(72.9 )	4.35/6.25(0 )	0.76/0.37 (48.68)
9190	211.86/134.46(36.53)	0.26/3.39(1288)	4.13/0.14(96.54)	717/3592(5.01)	80.16/3.56(95.56)	0.18/0.02(87.01)	79.98/3.53(95.58)	0.75/0.34 (45.33)
9191	2588.93/84.14(96.75)	0.32/0.33(101 )	26.53/0.77(97.1 )	571/881(1.54)	79.39/43.16(45.63)	0.25/0.07(70.76)	79.14/43.09(45.56)	10.56/0.28 (2.65)
9192	58.01/0.72(98.76)	0.24/4.83(2057)	7.85/0(99.99)	576/6768(11.74)	11.65/0.19(98.35)	0.13/0.02(87.54)	11.52/0.18(98.47)	0.06/0 (0)
9194	284.21/4.6(98.38)	0.19/0.32 (168)	27.34/0.05 (99.83)	484/6381 (13.16)	12.38/0.24(98.08)	0.2/0.03(83.79)	12.18/0.2 (98.32)	1.08/0 (0)
9195	3905.54/21.29(99.45)	0.36/6.2(1721)	33.5/0.06(99.81)	889/3161(3.55)	6.01/0.48(91.98)	0.14/0.05(64.99)	5.87/0.43(92.63)	16.13/0 (0)
Average	1209.5/67.79(75.34)	0.28/3.01(1056.12)	16.89/0.2(82.21)	634/3818(6.01)	32.34/8.99(71.6)	0.17/0.04(77.83)	32.17/8.95(71.76)	4.89/0.33(16.11)
T statistic	4.897	8.132	3.137	5.417	3.454	3.612	3.116	2.912
*P* value	0.004	0.000	0.026	0.006	0.018	0.015	0.026	0.033

**Notes.**

F_uric acid_ faecal uric acid P_uric acid_ Plasma uric acid Cl_uric acid_ Uric acid clearance calculated from plasma and faecal uric acid levels AUC_uric acid_ area under the uric acid plasma curve Cl_PAH_ PAH clearance Cl_iohexol_ iohexol clearance Tm_PAH_ maximum tubular transport rate of PAH Tm_(uricacid)_ maximum tubular transport rate of uric acid

Highlighted cells are the results for the two birds that died. Values are presented for the birds as healthy/diclofenac exposed with the percentage change in parenthesis. *P*-values represent the difference between the two phases.

### Radiography

**Figure 2:**
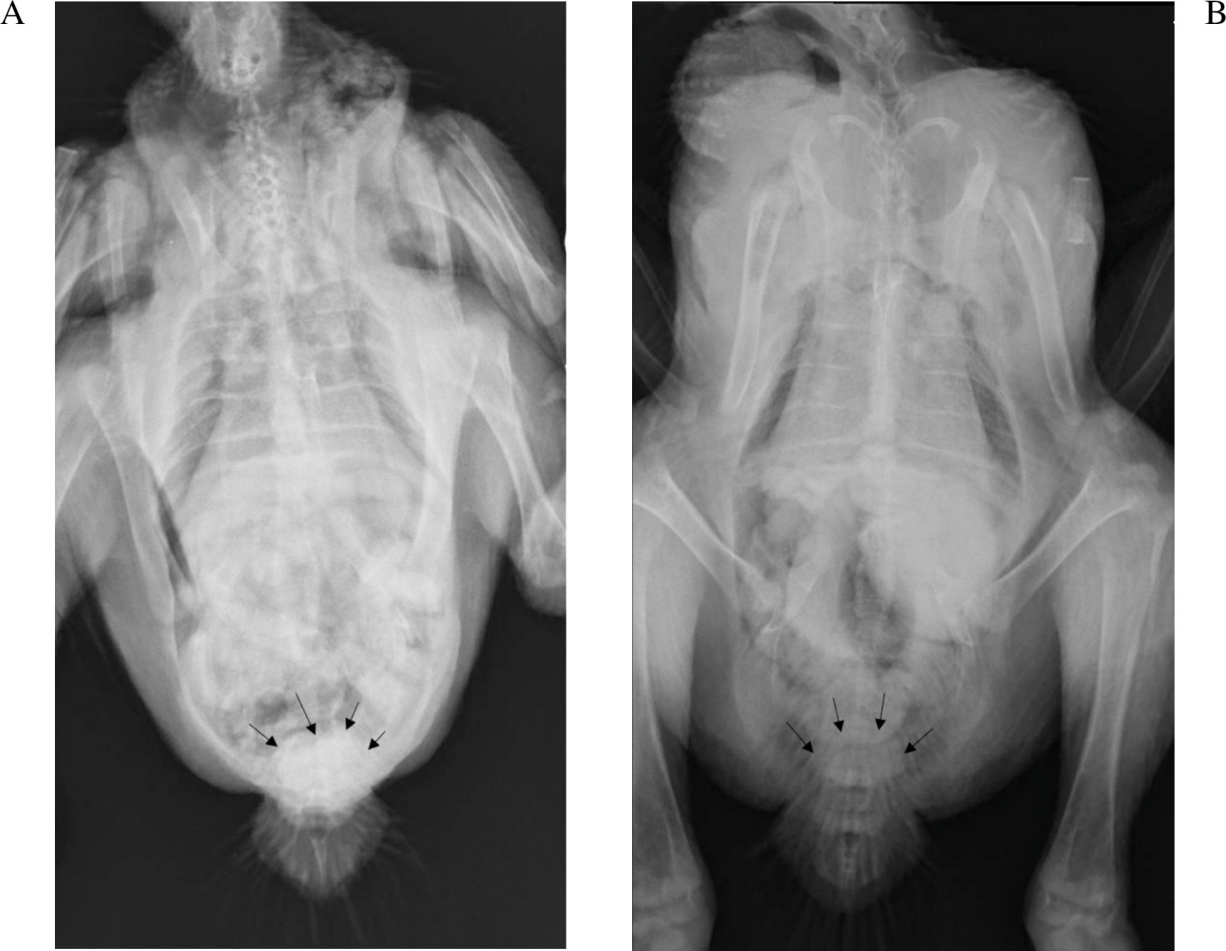
Radiographs of chicken 9192 five minutes after iohexol administration. The iohexol is more dense in the cloaca (arrows) in the absence of diclofenac (A) than with (B).

The kidneys were not visible in any of the chickens irrespective of the phase of the study, which was not unexpected since the kidneys are firmly attached to the renal fossae (Havenga, 2016). The IOH was already evident in the cloaca after 5 min. While subjective, the amount of IOH appeared to present at a lower concentration in the cloaca of the chickens in phase 2, 5 min after its administration with exception of 9189. The results for chicken 9192 which died were most clearly evident as seen in Fig. 2.

### Feeding Results

**Figure 3:**
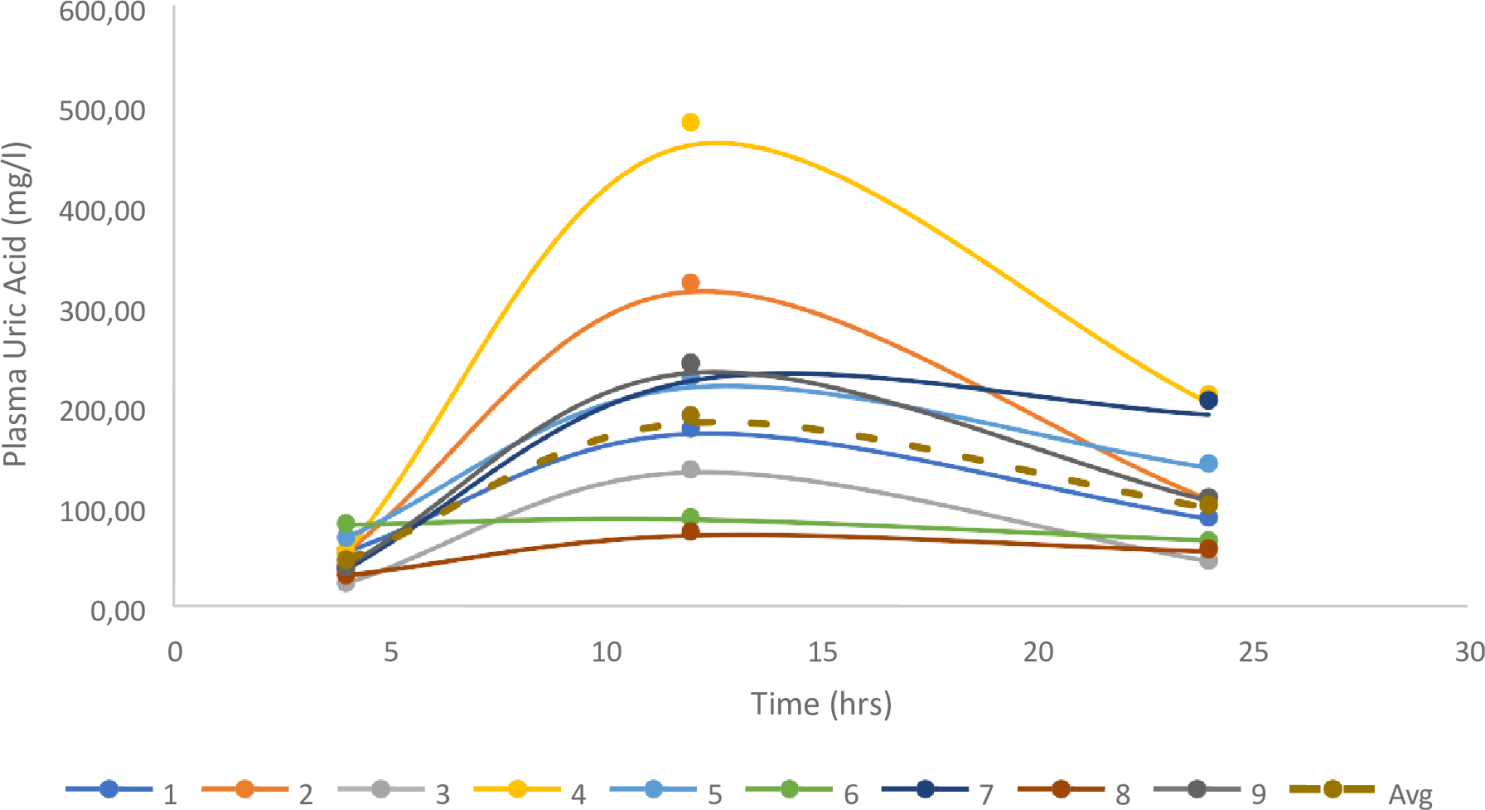
Individual changes in uric acid for the birds fed 200g of meat. The geometrical mean and Standard Error are also presented.

Following complete consumption of the offered meal, nine vultures had quantifiable levels and were included in the analysis. The two vultures without changes in uric acid did not consume the meal offered, and, while unintentional, gave a good indication of the basal uric acid change over the 24 h. For the vultures in which uric acid changes were evident, uric acid clearance was 1.88 ± 0.33 ml/min, based on the 200 g of meat, being equivalent to a 324 mg dose of uric acid, once again supporting lower uric acid clearance in a second vulture species (Fig. 3).

## Discussion

Diclofenac toxicity in the vultures and chickens is characterized by major increases in plasma uric acid and subsequent gout (Oaks et al., 2004; Meteyer et al., 2005; Reddy et al., 2006; Naidoo et al., 2007; Jain et al., 2009). From mammalian physiology it is known that diclofenac has the ability to inhibit both the functions of the OAT channel, reduce blood supply to the kidney and alter glomerulus filtration (Khamdang et al., 2002; Anzai, Kanai & Endou, 2006; Katzung, Masters & Trevor, 2009). However, unlike in mammals where glomerular filtration of uric acid is very efficient, birds excrete more than 80% of their total uric acid using the OATs channels (Berger, Yü & Gutman, 1960). This aligns with the findings of this study during which five chickens were excreting more than 85% of their total uric acid using former channels, except for one chicken that was somewhat lower at 64.24%. Furthermore, for the efficient plasma clearance of uric acid by the OAT channels, adequate blood supply needs to be maintained to the renal tubules (Khamdang et al., 2002; Anzai, Kanai & Endou, 2006; Katzung, Masters & Trevor, 2009). Previous studies have shown that the glomerulus received only arterial blood supply, while the avian renal tubules received an equal admixture of venous and arterial blood as ascertained through PAH clearance studies (Shideman et al., 1981). With diclofenac known to inhibit both tubular excretion and blood supply, both these mechanisms could explain the diclofenac induced increase evident in the chicken and vulture. Specifically, for this study we investigated the pathophysiology of diclofenac’s toxicity using IOH and PAH as marker substances. PAH is a substrate for OAT channels which are located on the basolateral membrane of the renal proximal tubules and IOH is a marker for renal blood flow and glomerulus filtration (Laroute et al., 2005; Sweet, 2005; Sweet, 2010; Van Wert, Gionfriddo & Sweet, 2010).

For this study we intentionally chose high doses as we wanted to follow the lethal changes following exposure to diclofenac. As expected, following the administration of 10 mg/kg diclofenac as a single dose, mortality rate of 33% was recorded within 48 h, which were similar to previous mortalities of 40% evident in white leghorn after 12 days of exposure to 10 mg/kg of diclofenac (Jain et al., 2009; Reddy et al., 2006) and the LD_50_ of 10 mg/kg reported after a single dose exposure in layer hens, and confirmed in Ross broilers of 4 weeks age (Naidoo et al., 2007; Locke, 2020). Furthermore, histopathology changes observed in the two domestic chickens that died following diclofenac exposure, were similar to those reported in the Leghorn and *Gyps* species (Oaks et al., 2004; Meteyer et al., 2005; Swan et al., 2006b; Jain et al., 2009; Naidoo et al., 2007). Likewise, the changes in uric acid concentration in these chickens were identical to that previously reported in vultures. It was also interesting to note that the one chicken which had mild urate induced renal necrosis did not succumb to toxicity within the 48 h monitoring period. This would imply that the difference observed in the former and other surviving chickens is subject to a degree of intra-subject variation which may be attributable to genetic, environmental and epigenetic factors (Fusco & Minelli, 2010; Frésard et al., 2013).

The half-life of elimination of diclofenac in the chickens was in all cases substantially shorter than previously reported in vultures (<2 h *vs* 14 h) (Naidoo & Swan, 2009). This was, however, very similar to the half-life of one hr reported in layer hens, albeit at a lower dose of 0.8 mg/kg (Naidoo et al., 2007). This would support previous reports that the unusual susceptibility of the vulture is related to metabolic constraints in the species (Hutchinson et al., 2014). Furthermore, in liver slice studies by Adawaren et al. (2018), the total protein content and thus cytochrome content, was deemed to be lower than in other avian species (Adawaren et al., 2018). Despite the short plasma half-life, the faecal half-life of elimination was in general longer. At present the exact reason for this is unknown, but we would speculate that the birds would have a degree of biliary retention as a result of the stress of being inducted into the study and single housed for the 24 h duration of the pharmacokinetic study (Harrison & Gibaldi, 1976). Also important to note, is that toxicity resulted a substantial time after diclofenac was no longer detectable in the plasma. This would likely indicate that the drug induced early cellular death, and that the changes in uric acid were likely a secondary outcome of toxicity. When comparing the chicken to the vulture, the maximum diclofenac plasma concentration for the vultures that died was 7.41 and 4.6 ug/ml respectively (Naidoo et al., 2009), which is above the toxic concentration of 4 µg/ml estimated for the chicken. This was evident despite the vulture being exposed to a dose of only 0.8 mg/kg as opposed to the 10 mg/kg in the chicken. Based on the difference in doses between the species, this would once again support metabolic constraint in the vulture, promoting plasma concentration that would inhibit uric acid excretion at a significantly lower dose.

The IOH clearance of 0.17 L/h*kg (2.83 ml/min*kg) for phase 1 of the study was similar to the IOH clearance of 2.94 ml/min*kg reported by Dhondt et al. (2019), which support the results from phase 1 being an accurate reflection of normal IOH clearance in healthy chickens. Following the administration of diclofenac, IOH clearance decreased in phase 2 of the study, indicating a decrease in glomerular filtration as IOH is purely dependent on glomerular filtration for its clearance. This finding was also in general supported by the radiographic analysis, which is a visual determination of glomerular filtration. This change in glomerular filtration rate (GFR) for chickens in phase 2, also indicates that diclofenac consistently decreased the blood supply to the kidneys in all chickens, and thus alters the rate of delivery of uric acid to the excretion points. This finding is not unusual as elderly people on oral doses of diclofenac (50 mg three times a day), were reported to have impaired renal blood flow and glomerular filtration rates leading to renal failure (Katzung, Masters & Trevor, 2009).This effect results from the suppression of the synthesis of prostaglandin through the non-specific inhibition of the cyclo-oxygenase enzyme in animals. Prostaglandins play a physiologic role in maintaining GFR. Prostaglandins predominately function to regulate renal blood flow primarily supplied *via* the afferent arterioles and as a renal vasodilator (Verlander, 1997; Oates et al., 1998). Using chicken venous tissue, Naidoo et al. (2007) were able to demonstrate that diclofenac was also a venodialatory substance. Based on the similarity between human and chicken COX2 enzymes, it is likely that COX inhibition drives this process in the chicken (Botting, 2006). It was also interesting to note that, due to the higher plasma concentration and despite the lower GFR, the predicted total uric acid filtered was higher than in phase 1 for some of the chickens.

For the four chickens in which PAH clearance was properly quantifiable in phase 1, their PAH clearance of 143.8 ml/min*kg compared well with Dhondt et al. (2019) who described PAH clearance of 117.5 ml/min*kg. The change for PAH noted in phase 2 was, however, not constant in the treated chickens and was very markedly changed in the two chickens that died with the decreased PAH clearance evident before 12 h, well-before the birds started showing clinical signs of toxicity at 24 h post-exposure. In terms of the general effect of diclofenac on PAH clearance, the finding was not surprising as both sodium salicylate and phenylbutazone have previously shown to inhibit PAH clearance in the chicken by 90 and 50% respectively, albeit at much higher doses (Berger, Yü & Gutman, 1960). The necropsy of these dead chickens further revealed urate deposits on kidney and other organs. Urate deposits were associated with very high plasma uric acid concentration which had multiplied more than 10-fold from its original concentration, reaching maximum levels of 6.196 mmol/l from 0.250 mmol/l in one chicken. With IOH clearance being constantly decreased in all the chickens, and PAH clearance being almost insignificant in only the two chickens that died (Tm_PAH_ and Cl_UA_ both reduced by more than 98%, furthermore with Tm_uricacid_ being absent), it appears that tubular excretion of uric acid was affected. In a study by Sun et al. (2021), using molecular techniques in the chicken cells, they were able to demonstrate that diclofenac likely induces kidney cell apoptosis, through interfering with purine metabolism and inhibiting the expression of OAT2. These factors in combination were speculated to induce increase the uric acid concentration in chickens leading to hyperuricemia and urate deposition.

Based on the overall evidence obtained from this study, it offers insight into the mechanism of toxicity. Firstly, with the short half-life of diclofenac, it is unlikely that the drug had a primary inhibitory effect on the functionality of the uric acid transporter system to such a large extent, as we initially speculated in our hypothesis. However, delayed effect of uric acid excretion has been described in humans, where the uricosuric effect of diclofenac was still evident a day after a single treatment despite the half-life of elimination circa 2 h, the effect on uric acid seen in people was not as marked as in this study (Davies & Anderson, 1997; Tiitinen et al., 1983). This would indicate that acute cellular (renal) toxicity and apoptosis is the likely underlying mechanism of toxicity, as is evident from the cellular death shown by the necropsy on the two dead birds. From previous published work this has been shown to result from reactive oxygen species (ROS) induced cellular death, 2 h after exposure under *in vitro* conditions (Naidoo & Swan, 2009), the same time period required for the PAH increase evident in this study together with reduced OAT2 expression (Sun et al., 2021). However, since only two birds died, a degree of individual variability in protection against ROS is likely to be present. To further understand this mechanism, further studies can focus on direct markers of cellular death such as gamma-glutamyl transferase (GGT), pro-inflammatory cytokines and creatinine (Mohan et al., 2012).

With decreased uric acid and PAH clearance being the predominant change following diclofenac exposure, the results from this study also offers some insight into the sensitivity of the vulture compared to the chicken. The uric clearance in the chicken was at minimum two-fold higher than the vulture despite the high protein diet of the vulture when corrected for body mass. In general, it is accepted that the main source of uric acid in protein diet is red meat especially beef, lamb, veal and pork (Teng et al., 2015). As is evident in the feeding study, after a protein meal plasma uric acid levels are raised for a fairly long period in the vulture. With tubular damage resulting in an increase in uric acid, it is not surprising that the vulture’s diet predisposes them to toxicity. However, the unexpected finding was the difference in the uric acid clearance between vultures and chickens. What makes this a surprising finding is that with the vulture, being a carnivore and naturally exposed to a high uric acid load, one would have expected a more efficient uric acid excretory system. This would then suggest that a secondary protection mechanism has to be present. Evaluating the digestive system, the crop would be the likely protective mechanism. As a structure the crop evolved to allow for large scale feeding (Zheng et al., 2011) and slow release of food in the birds which—in the African White- Backed Vulture - was established as a 28 h period (Kierończyk et al., 2016). This lower rate of release would create the buffer, akin to a valve, allowing for slower increases in plasma uric acid concentration which would not exceed renal excretory capacity. The lower ability of the kidney to excrete uric acid is also likely to be the pitfall of not needing to adapt to smaller more frequent meals like other species with rapid GIT transit (McWhorter, Caviedes-Vidal & Karasov, 2009). Unfortunately, the true effect of the full crop on plasma uric acid concentrations could not be measured as fully engorged birds regurgitate as a natural defense mechanism when handled.

## Conclusion

Diclofenac administration, together with IOH and PAH in the chickens allowed for real-time evaluation of changes. In untreated chicken, no ill heath was noted but in treated chicken, within 48 h, 33% of the chickens died and were thereafter diagnosed with urate nephropathy. IOH clearance of the untreated birds was fast and most cleared within 6h. However, for the treated group uniform IOH clearance decreased by up to 87%, leading to a conclusion that diclofenac interferes with renal arterial blood flow with resultant decreased glomerular blood supply. PAH clearance of the untreated birds was rapid and non-detectable with up to 99.90% excreted while for the treated birds, diclofenac induced an increase in the total exposure to PAH. Death, however, only results when there is near 100% inhibition of uric acid excretion from total glomerular and tubular inhibition. It should also be noted that toxicity occurred in the relative absence of diclofenac in plasma, whilst the drug was present in vultures for over 14 h. It should be noted that the following study intended to only look at the lethal changes induced by the drug. It is possible that lower doses, in the absence of overt toxicity, may have induced more subtle changes in physiological functioning, which may require further elucidation.

##  Supplemental Information

10.7717/peerj.12002/supp-1 Supplemental Information 1 Raw data

10.7717/peerj.12002/supp-2 Supplemental Information 2 Check list

10.7717/peerj.12002/supp-3 Supplemental Information 3 Suppplemental figures

## Additional Information and Declarations

### Data Availability

The following information was supplied regarding data availability: Raw data are available as Supplemental Files.
