# Supplementary material for: Diclofenac toxicity in susceptible bird species results from a combination of reduced glomerular filtration and plasma flow with subsequent renal tubular necrosis
Source: PeerJ. 2021 Aug 23;9:e12002. doi: 10.7717/peerj.12002 (PMC8388555; doi:10.7717/peerj.12002)
Supplement: Supplemental Information 3 [file peerj-09-12002-s003.docx]

**The influence of diclofenac on P-aminohippuric acid and iohexol transport in chickens, as a further study on the toxicity of the drug in vultures.**

B Nethathe^1,2^, J Chipangura^2^, IZ Hassan^1^, N Duncan^3^, EO Adawaren^1^, LN Havenga^4^, V Naidoo^1^

^1^ Department of Paraclinical Sciences, Faculty of Veterinary Science, University of Pretoria, Pretoria, South Africa.

^2^ Department of Food Science and Technology, School of Agriculture, University of Venda for Science and Technology, Limpopo, South Africa

^3^ Department of Pathology, Faculty of Veterinary Science, University of Pretoria, Pretoria, South Africa.

^4^ Department of Anatomy and Physiology, Faculty of Veterinary Science, University of Pretoria, Pretoria, South Africa.

Corresponding author: Bono Nethathe^1^


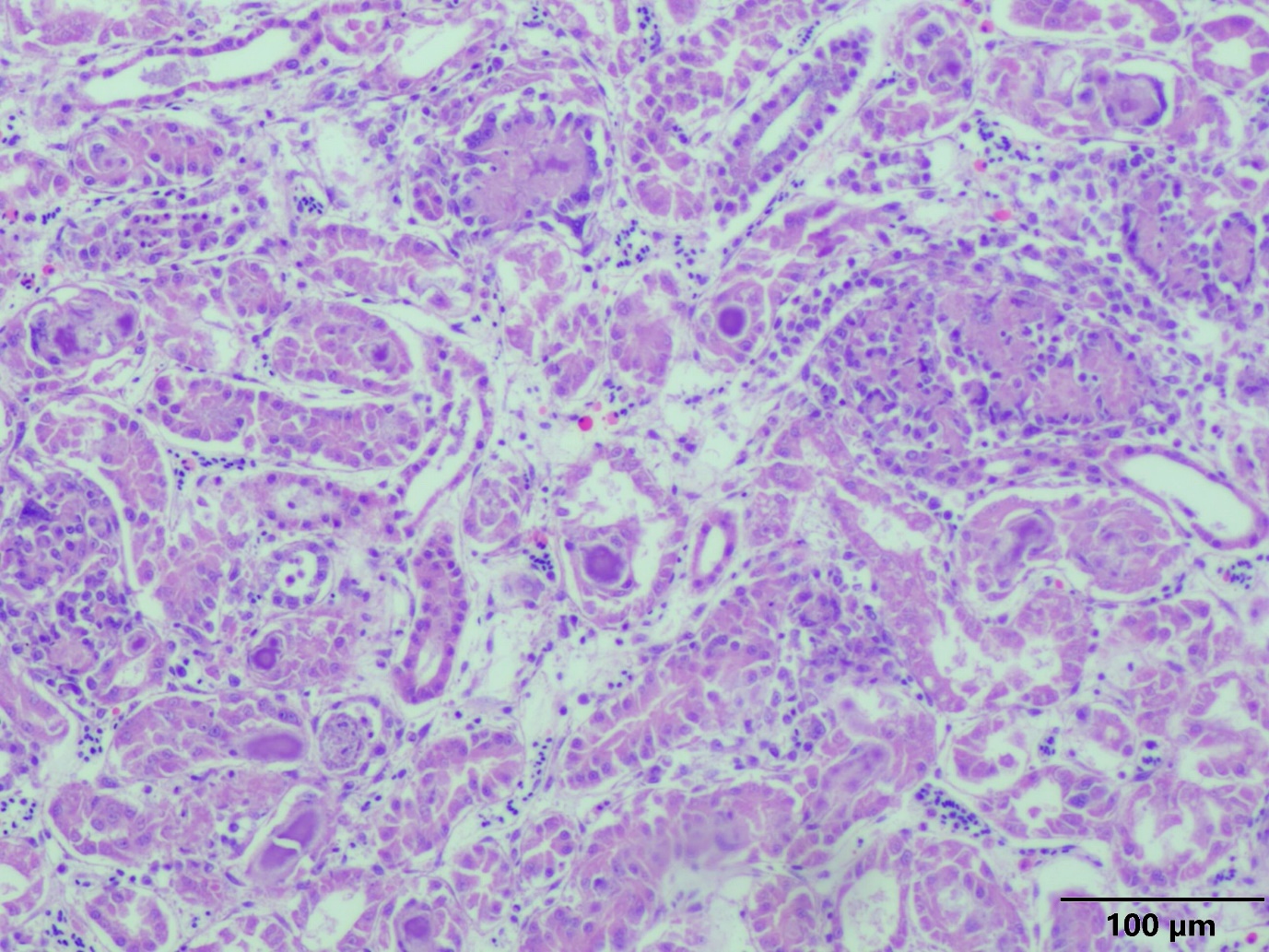


Supplementary Fig 1: Kidney (10x magnification). The picture shows scattered purple, pink globular urates within renal tubules. Also visible are dilated tubules containing sloughed degenerated renal tubular epithelial cells. Glomeruli have been replaced by amorphous pink mass of urates surrounded by macrophages.


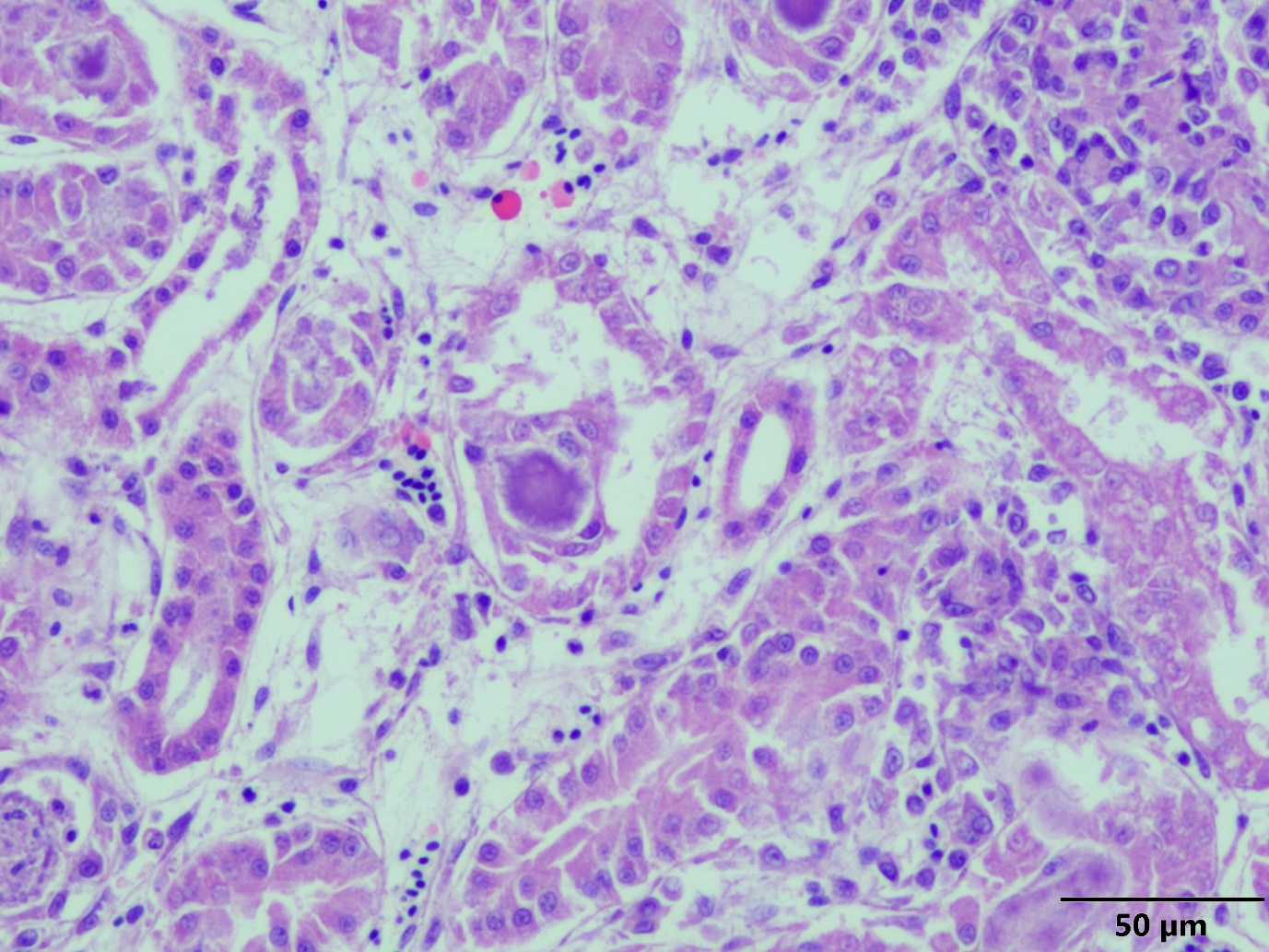


Supplementary Fig 2: Kidney (20x magnification). Shows urates filling the tubule lumen.


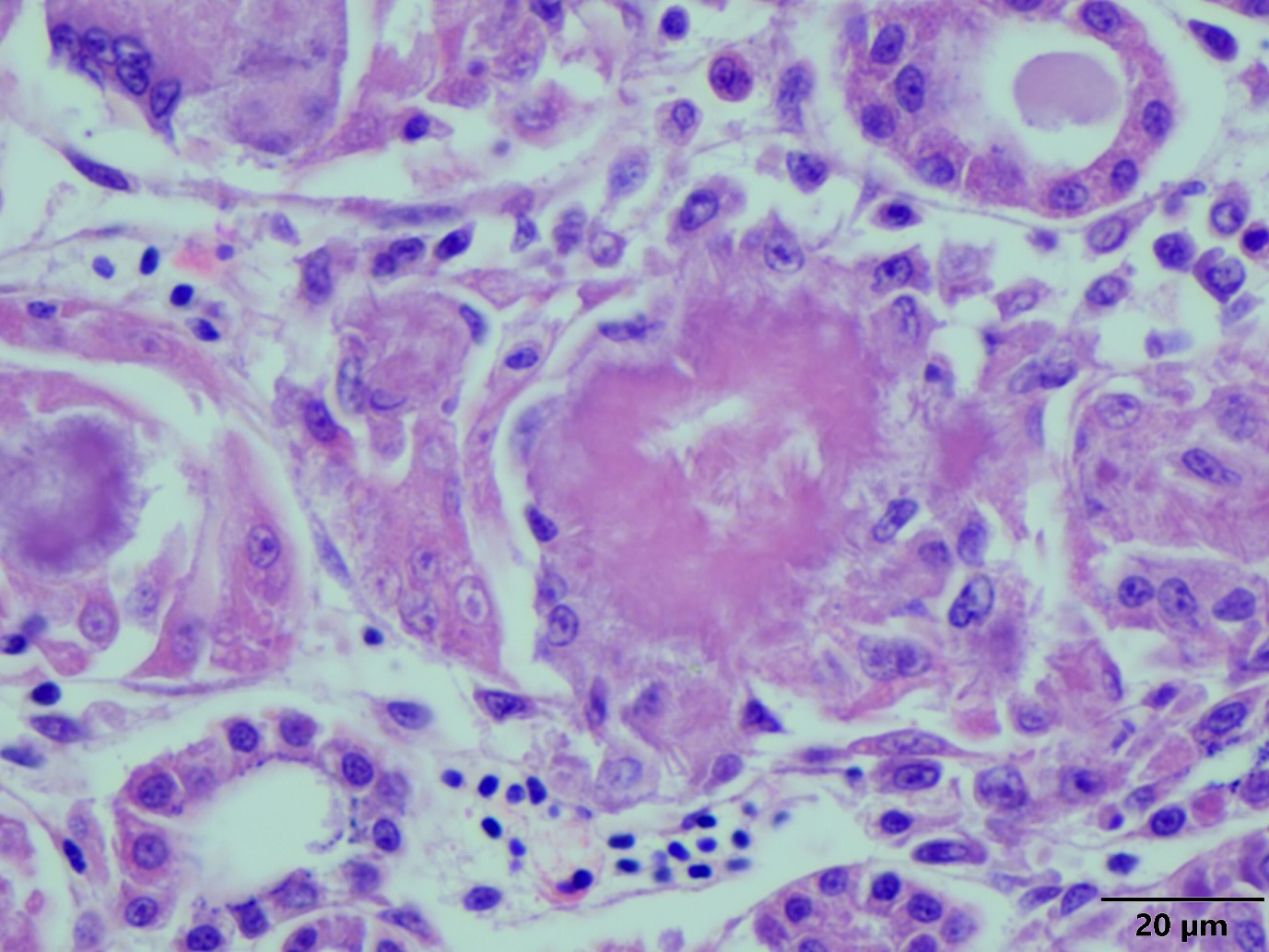


Supplementary Fig 3: Kidney (40x magnification). Spicules of uric acid can be seen filling the renal tubule lumen.


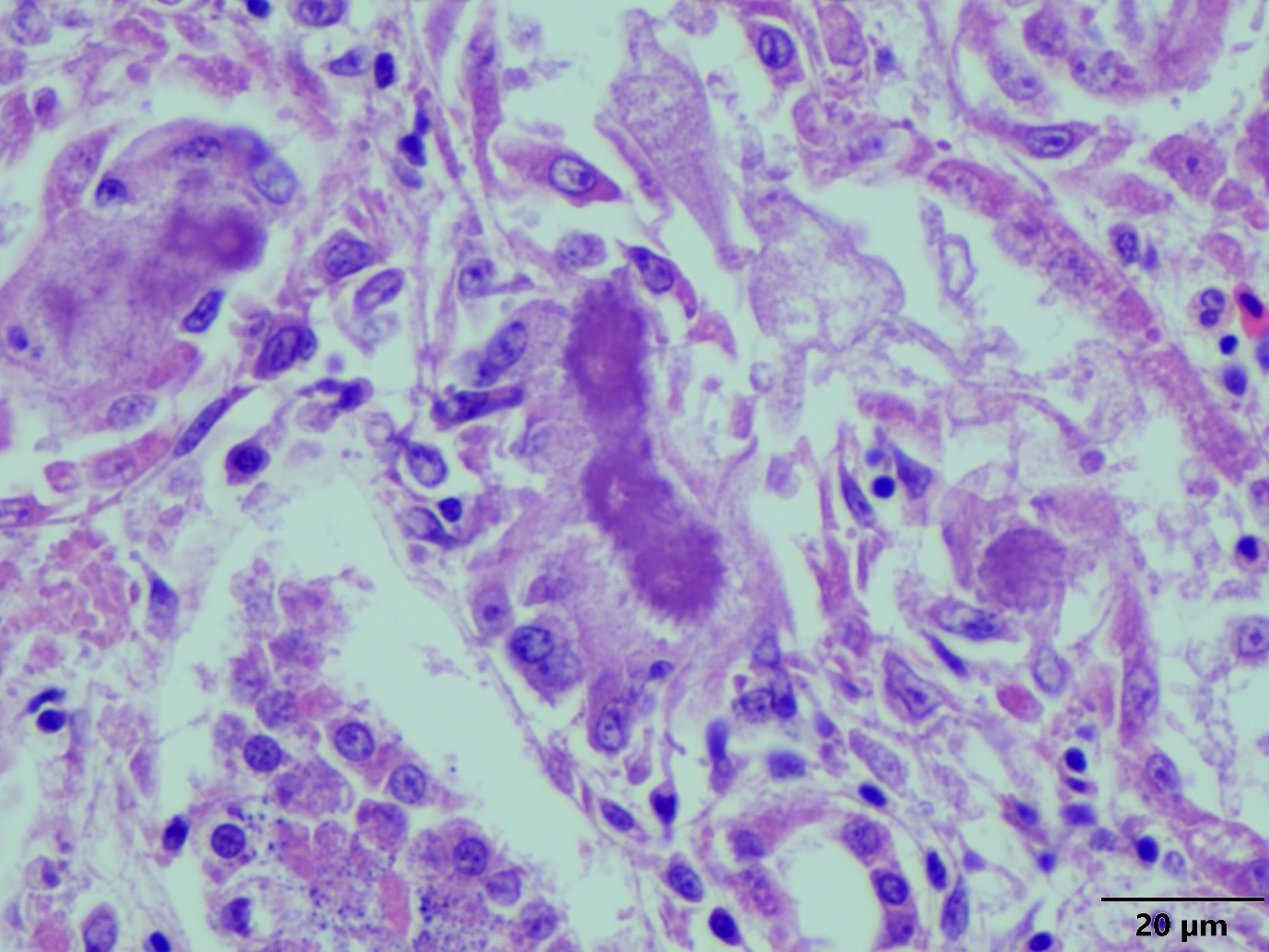


Supplementary Fig 4: Kidney (40x magnification). Urates filling a renal tubule. Also evident is the loss of scattered renal tubular epithelial cells around the periphery.

Supplementary Fig 5: Diclofenac plasma vs time profile for bird 9189 and 9190 following dosing at 10 mg/kg (Sampling points based on PAH administration times).

**B**


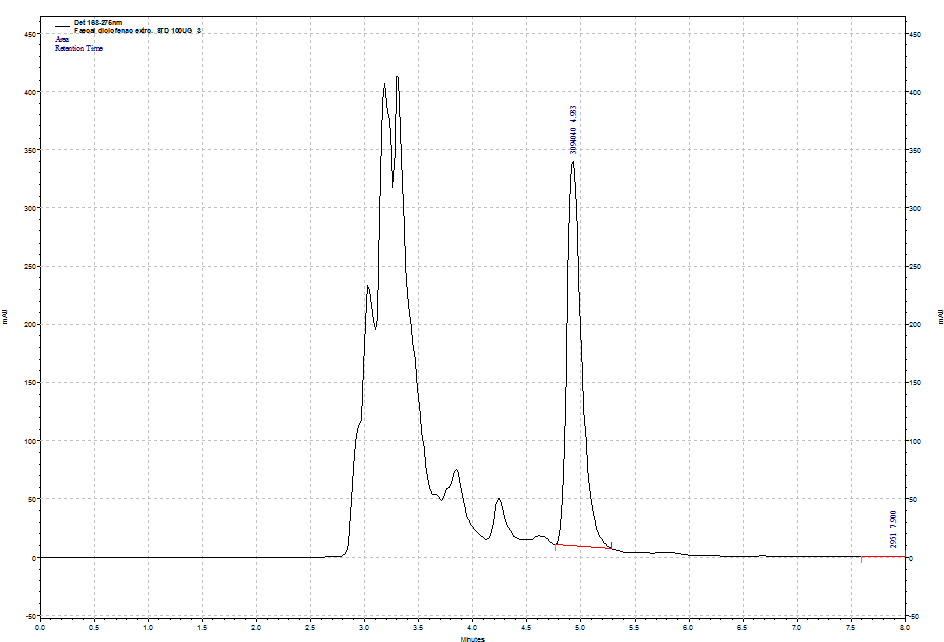


Diclofenac

Supplementary Fig 6: A) Representative of chromatogram of pure diclofenac standard and B) spiked faecal sample with 100 µg/ml of diclofenac
